# Effect of Temperature on the Composition of a Synthetic Hydrocarbon Aviation Lubricating Oil

**DOI:** 10.3390/ma13071606

**Published:** 2020-04-01

**Authors:** Zhuoting Gan, Ting Yao, Meng Zhang, Jianqiang Hu, Xiaoxiao Liao, Yongli Shen

**Affiliations:** 1 School of Tourism, Huangshan University, Huangshan 245041, China; 2 Analysis and Test Center, Huangshan University, Huangshan 245041, China; 3 Department of Aviation Oil, Air Force Logistics College, Xuzhou 221006, China; 4 Unit 94923 of People’s Liberation Army of China, Wuyishan 354301, China

**Keywords:** synthetic hydrocarbon aviation lubricating oil, high temperature, composition, structure, advanced polymer chromatography, cluster analysis

## Abstract

Synthetic hydrocarbon aviation lubricating oils (SHALOs) gradually degrade over time when subjected to high temperatures, resulting in their composition and properties varying over the operation lifetime. Therefore, understanding the SHALO degradation properties by elucidating the mechanism on a molecular level, as a function of high temperature, is of interest. A SHALO was subjected to thermal treatment (TT) at 180, 200, 230, 250, 270, or 300 °C for 2 h. The chemical compositions of six TT samples and one fresh oil were analyzed by fourier transform infrared F spectroscopy, advanced polymer chromatography, and gas chromatography/mass spectrometry. Furthermore, the physicochemical properties, such as kinematic viscosity, pour point, and acid number, of seven samples were determined. The oil samples were grouped by cluster analysis (CA) using a statistical method. The SHALO was identified to comprise 20 functional groups, including comb-like alkanes, long-chain diesters, amines, phenols, and other compounds. TT at <230 °C caused partial cracking of the SHALO base oils, with a concomitant change in the antioxidant content and type, and the polycondensation reactions were dominant. The observed antioxidant changes were not obvious from TT at >230 °C. A large number of small-molecule compounds were detected, including n-alkanes and olefins. TT at 250 °C was shown to be an important threshold for the kinematic viscosity, pour point, and acid number of the samples. Below 250 °C, the sample properties were relatively stable; but at elevated TT temperatures (>250 °C), the properties were observed to dramatically degrade. As the sample color was highly sensitive to temperature, the TT temperature induced rapid and significant color changes. The CA analysis results for the oil compounds at the molecular level were in good agreement with observed changes in the physicochemical properties at the macro level.

## 1. Introduction

Synthetic lubricating oils (SLOs) are liquid lubricants, possessing specific structures and properties derived from organic synthesis. Compared with vegetable oils and mineral-based lubricating oils, SLOs have the advantages of eco-compatibility, excellent thermal stability, and low-temperature behavior [1,2]. There are six types of SLOs, namely, synthetic hydrocarbon oils, organic acid ester synthetic oils, polyether synthetic oils, phosphate esters, fluorine-containing oils, and silicone oils. The SLO types exhibit significant differences in chemical structure, lubricating properties, and antidegradation behaviors compared with the base oil. Therefore, specific SLOs are better suited to different working conditions. Hydrocarbon and ester synthetic oils are often employed in the military, aerospace industry, and under other harsh working conditions owing to their anti-high temperature performance [3].

The base oil is the principal component of an SLO, accounting for 70–90 wt.%, and is not only the carrier of lubricating additives but also directly determines the lubricating performance [4]. The base oil of synthetic hydrocarbon aviation lubricating oil (SHALO) is synthesized from ethylene as the raw material, which is first polymerized into an α-olefin, followed by polymerization to afford a poly-α-olefin (PAO), or obtained by copolymerization of PAO with ethylene. PAO has a unique comb-like hydrocarbon structure that enables SHALO to possess excellent viscosity-temperature properties, low-temperature fluidity, low volatilization loss, and good thermal and hydrolysis stability. Furthermore, the PAO properties enable SHALO to exhibit excellent oxidation, shear, and low corrosion properties, as well as excellent lubrication and wear properties [5,6].

With the development of aero-engines designed to improve thrust-to-weight ratios while maintaining high reliability and durability, the operating conditions that lubricants are subjected to are becoming increasingly complicated and demanding [7]. When in use, the temperature near to the first piston ring of the main bearing can reach 200–300 °C [8]. High temperatures are considered a critical factor in the acceleration of lubricant degradation, which might impact flight safety under harsh conditions [9,10]. Recently, much interdependent research has focused on the effect of temperature on the degradation of synthetic aviation lubricants, with respect to lubricating properties and the associated mechanisms [11,12,13].

Research has shown that high temperatures accelerate the cracking, oxidation, and polymerization reactions of SHALO [14,15] while also promoting the desorption of lubricants that negatively impact directional adsorption, melting the oil film [16], and reducing the anti-foaming performance [17]. Furthermore, carbide accumulation [18,19,20], the blockage of fuel supply lines and filtration systems [21], and reduced thermal conductivity of the engine are often observed during oxidation at high temperatures. Therefore, tight and stagnant moving joints [22,23] form, resulting in reduced engine operation and service life. Although some reports have demonstrated that high temperatures reduce the lubricating properties of SHALO, other studies have observed improved friction properties after the appearance of oxidized derivatives in aged oils [24]. Therefore, elucidating the SHALO lubricating properties as a function of engine warming during aircraft flight is required.

The present study also aimed to demonstrate how changes in the SHALO molecular structure and base oil composition through organic synthesis design improve the lubricating properties of lubricating oils and increase compound stability. Changes to the compound structures and SHALO compositions are the principal reason for the degradation of lubricating properties. Furthermore, changes in the molecular structure and composition as a function of the heating process need to be elucidated at the molecular level.

The gas-phase thermal degradation of three commercial industrial lubricants (two different triaryl phosphate compositions and one based on fatty acid methyl and ethyl esters) was investigated under oxidative pyrolysis conditions between 400 and 1000 °C by Mascolo et al. [25]. Significant differences were observed in the thermal stability between lubricants containing phosphorus and those without phosphorus. Lubricant degradation in the absence of phosphorus was assumed to start with the breakdown of aliphatic moieties to generate vinyl radicals, which, in turn, afforded benzene radicals and polycyclic aromatic hydrocarbons (PAHs) through displacement and cyclization reactions. However, phosphorus-based lubricants degraded to directly form benzene radicals and PAH structures through consecutive reactions. At temperatures of >700 °C, the benzene and PAH contents in the degradation products of lubricants without phosphorus were significantly lower than in corresponding lubricants containing phosphorus [25]. Various types of synthetic lubricants are observed to undergo different degradation processes.

Tripathi and Vinu [26] observed that the major hydrocarbon present in fresh synthetic oil was tetratriacontane (C_34_H_70_) and that the oxidative degradation of synthetic oil as a function of temperature resulted in the formation of shorter-chain hydrocarbons as the major products. For example, shorter-chain hydrocarbons, such as pentadecane (C_15_H_32_) and hexadecane (C_16_H_34_), were the major products at 200 °C, while longer-chain hydrocarbons, such as tetrapentacontane (C_54_H_110_) and hexacontane (C_60_H_122_), were observed along with other hydrocarbons at 120 and 149 °C, respectively. The chemical reactions of synthetic lubricants during heating are complicated, with cracking and polymerization phenomena occurring simultaneously. The oxidation mechanism of SHALOs is generally considered to occur via a radical chain mechanism, in which the hydrocarbons in the lubricating oil are pyrolyzed to form organic free radicals that react with oxygen to form peroxy radicals and peroxides, and thereafter, polymerize to form oligomers [21,27,28]. However, Wu et al. [15] subjected synthetic lubricating oil diethylhexyl sebacate (DEHS) to continuous high-temperature treatment (150 °C) and observed that DEHS degradation not only resulted in oxidation reactions but also hydrolysis reactions. Furthermore, Santos et al. [29] observed that the content of insoluble high-molecular-weight polymers in mineral lubricating oils increased as a function of temperature, together with an increase in the kinematic viscosity (KV) of the lubricating oils. Meanwhile, computational methods are valuable tools for determining properties that are dependent on the microstructure. Gueorguiev et al. developed the synthetic growth concept based on density functional theory, with their computational experiment providing useful information to achieve a systematic understanding of the stability/reactivity and identification of the various compounds [30,31]. Furthermore, the thermal oxidation process of lubricating oils has been simulated using visual reactive force field molecular dynamics at an atomic level [9,13]. Typically, the degradation process of lubricating oils at high temperatures is relatively complicated from a molecular perspective. The influence of temperature on the structure and composition of lubricating oils still requires elucidation, while structural and compositional changes during the heat treatment of lubricating oils, especially SHALOs, remain unclear.

Based on actual working conditions, SHALO was subjected to thermal treatment (TT) at six different temperatures. The six TT samples and one fresh oil were analyzed by fourier transform infrared spectroscopy (FT-IR), advanced polymer chromatography (APC), and gas chromatography/mass spectrometry (GC/MS) to explore changes in the SHALO composition and structure in more detail. Furthermore, the degradation mechanism and physicochemical properties were described, including KV, pour point (PP), acid number (AN), and color. This study aimed to develop an understanding of the SHALO macroscopic-microscopic structure relationship and to elucidate changes in the composition and physicochemical properties as a function of the TT process.

## 2. Experimental

### 2.1. Materials

The SHALO sample was supplied by Unit 94923 of the People’s Liberation Army of China. SHALO comprised base oils, which are low-viscosity PAOs, together with nominal amounts of esters (ETs) and additives, such as antioxidants, antifoaming agents, and preservatives. The SHALO KV at 100 °C was ≥3 mm^2^/s, the AN was ≤0.05 mg KOH/g, and the specified operating temperature range was −40 to 200 °C. All solvents used in experiments were commercially purchased analytical reagents and purified by distillation prior to use.

### 2.2. Sample Preparation

SHALO (150 mL) was placed into a 500-mL stainless-steel autoclave and heated to 180, 200, 230, 250, 270, or 300 °C with magnetic stirring at 1000 rpm, and each temperature was maintained for 2 h, followed by immediately cooling of the reactor to room temperature in an ice-water bath within 0.5 h. The TT samples were obtained from the autoclave by filtration through a polytetrafluoroethylene membrane (pore size, 0.45 μm) and denoted as S_180_, S_200_, S_230_, S_250_, S_270_, and S_300_, respectively. The untreated oil control sample was denoted as S_rt_.

### 2.3. Sample Analysis

All SHALO samples were characterized by FT-IR spectroscopy and APC. FT-IR spectra were recorded using an FT-IR spectrometer (Nicolet 380, Thermo Fisher, Waltham, MA, USA) across a wavenumber range of 400–4000 cm^−1^. Each sample was mixed with KBr and pressed into a pellet for FT-IR analysis. APC analysis was performed using a GC system (ACQUITY APC, Waters, Milford, MA, USA) equipped with three ACQUITY APC XT columns placed in series, as follows: 450 Å column (4.6 × 150 mm, 2.5 μm); 200 Å column (4.6 × 150 mm, 2.5 μm); 45 Å column (4.6 × 150 mm, 1.7 μm). The ranges of effective molecular weights of each column were 20,000–400,000, 3000–70,000, and 200–5000, respectively. The columns were kept at 45 °C. Tetrahydrofuran (THF) was used as the mobile phase at a flow rate of 0.5 mL/min. Each sample (5 mg) was diluted to 1.5 mL with THF. The injection volume was 10 μL.

All samples were analyzed by GC/MS using a GC/MS system (Hewlett-Packard 6890/5973, Agilent, Santa Clara, CA, USA) equipped with a capillary column coated with HP-5MS (30 m × 0.25 m inner diameter, film thickness of 0.25 μm, cross-linked 0.5% PhMe siloxane). The injector temperature was set at 300 °C, with high purity helium used as the carrier gas. The electron impact mode ion source temperature was set at 230 °C, with the electron energy set at 70 eV, and a mass range of m/z 35–500. The initial oven temperature was 120 °C, which was increased to 274 °C at a rate 13 °C min^−1^, then to 281 °C at a rate of 0.5 °C min^−1^, holding for 2 min, and finally to 300 °C at a rate of 12 °C min^−1^, holding for 5 min. Data acquired was processed using MSD ChemStation software (D.02.00, Agilent, Santa Clara, CA, USA). Compounds were identified by comparing the mass spectra with NIST11 library data. The relative content of each compound was determined by peak area normalization. Compounds were analyzed using Origin Pro 2018 software (OriginLab, Northampton, MA, USA) for cluster analysis (CA).

The physicochemical properties were determined simultaneously. The color was determined using a visual method (GB/T6540-1986) [32], KV was measured using a glass capillary viscometer (GB/T 265/1988) [33] at 40 °C, AN was determined using a glass indicator electrode method (GB/T7304-2000) [34], and PP was determined using a PP method (GB/T3535-2006) [35].

## 3. Results

### 3.1. Subsection FT-IR Analysis

**Figure 1:**
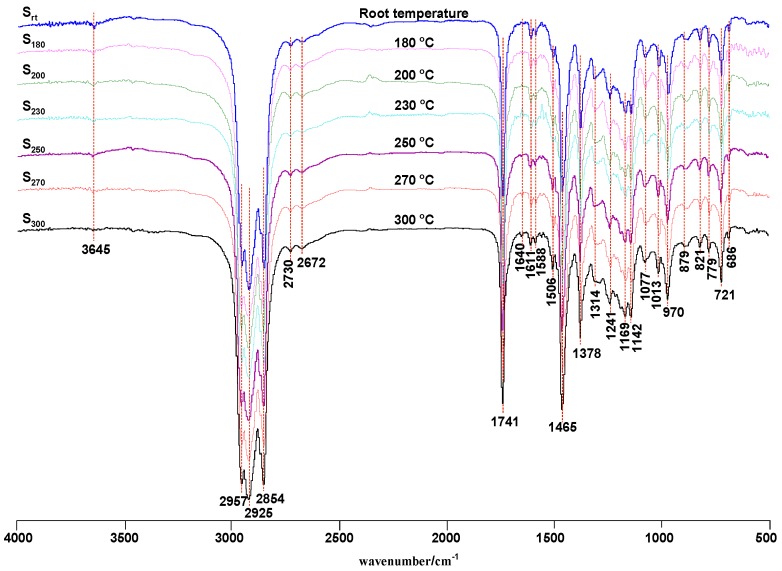
FT-IR spectra of S_rt_, S_180_, S_200_, S_230_, S_250_, S_270_, and S_300_.

As shown in Figure 1, there were no significant differences between the S_180_–S_300_ and S_rt_ FT-IR spectra. Twenty functional groups were detected in the FT-IR spectra (Table 1). Some functional groups exhibited absorbances attributed to aliphatic moieties at ~2854, ~2925, and 2957 cm^−1^, C=O vibrations at ~1741 cm^−1^, CH_2_ vibrations at ~1465 cm^−1^, –CH(CH_3_)_2_ vibrations at ~1378 cm^−1^, =CH–H vibrations at ~970 cm^−1^, and –(CH_2_)_n_– vibrations at ~720 cm^−1^, which were significantly stronger than others, implying that the components in the oil samples mainly comprised these groups.

The bands at 2957, 2925, and 2854 cm^−1^ were attributed to aliphatic –CH_3_ and –CH_2_ stretching vibrations, the band at ~1465 cm^−1^ was attributed to –CH_2_ shear bending vibrations, and the band at 1378 cm^−1^ was assigned to the C–H deformation vibration in –CH(CH_3_)_2_. A sharp and strong band was observed at 721 cm^−1^, implying that samples were rich in four or more –CH_2_ moieties. Functional groups detected in the samples indicated an abundance of long-chain aliphatic species.

The peak at 1741 cm^−1^ was assigned to the C=O stretching vibration in saturated fatty acid esters (ν_C=O_). The peaks at 1169 and 1142 cm^−1^ were attributed to the C–O–C stretching vibrations in long-chain fatty acid esters (ν^as^_(COC)_), suggesting an abundance of long-chain fatty acid esters present in the sample.

The weak peaks located at ~2730 and ~2672 cm^−1^ were assigned to the –CHO stretching and C–H bending vibrations of the aldehyde group, respectively. The bands located at ~1611 and ~1588 cm^−1^ were attributed to aromatic ring skeletal vibrations (ν), while those at ~879, ~821, and ~779 cm^−1^ corresponded to o-, m-, and p-substituents on the aromatic ring, respectively. The peak at 1506 cm^−1^ was assigned to the N–H bending vibrations (ν_NH_) in secondary amines. The C–N stretching vibrations (ν_C–N_) of aromatic secondary amines produced bands at 1241 and 1314 cm^−1^. The peaks observed at 3645 and 686 cm^−1^ were attributed to O–H stretching vibrations (ν_OH_) and O–H curling vibrations (τ) in phenols, respectively. These data demonstrated that SHALOs were mixed with base oils comprising long-chain alkanes, saturated fatty acid esters, and additives, such as amines and phenols.

As shown in Figure 1 and Table 1, the FTIR spectra of the seven oil samples changed very little under high-temperature treatment. Strong absorbances at around 2957, 2925, 2854, 1741, 1465, 970, and 721 cm^−1^ suggested that long-chain alkanes, carbonyl compounds, and unsaturated hydrocarbons were still abundant in the aged oils. The O–H stretching vibration located at ~3645 cm^−1^ decreased as a function of TT temperature until the vibration was no longer detected, indicating that phenolic antioxidants were continuously consumed. The C=C stretching vibration observed at ~1640 cm^−1^ was only detected in S_300_, which suggested that long-chain hydrocarbons were broken into olefins (OFs) and other substances as a function of TT temperature. Thermal cracking and polymerization reactions were concluded to occur to varying degrees during the high-temperature treatment of SHALOs, with antioxidant consumption somewhat preventing the lubricating oil properties from diminishing.

### 3.2. APC Analysis

The relative molecular mass and corresponding mass distribution are among the most basic structural parameters of lubricating oils that are closely related to the physicochemical properties (such as KV and PP) and the thermal and tribological properties of the lubricating oil. The molecular mechanism of thermal stability can be explored using the molecular weight and corresponding mass distribution. APC is an effective method for measuring the molecular weight and molecular weight distribution of lubricating oils. APC can also obtain important molecular weight information, such as the weight-average molecular weight (M_w_) and number-average molecular weight (M_n_).

**Figure 2:**
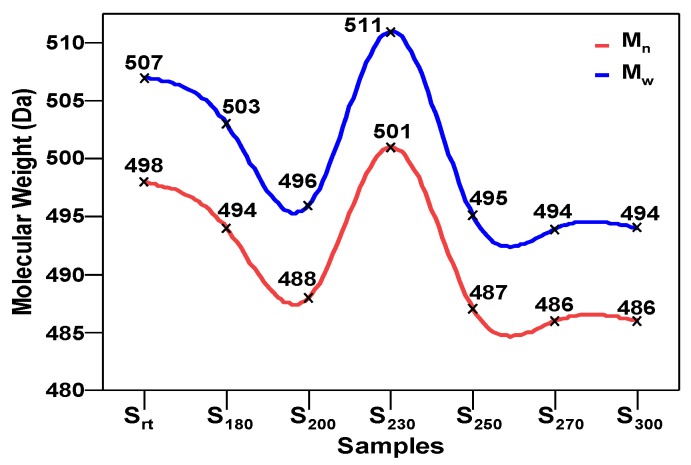
Molecular weight distributions of S_rt_, S_180_, S_200_, S_230_, S_250_, S_270_, and S_300_.

**Figure 3:**
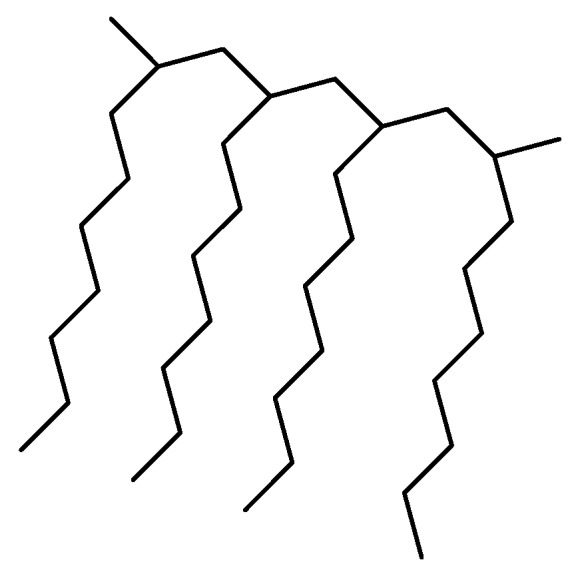
The possible structural formula of the poly-α-olefin (PAO).

The SHALO used in this study contained a base oil comprising 80% PAO, 20% diisooctyl adipate (DIOA), and a small number of additives. A previous study [36] has shown that PAO is polymerized and comprises linear α-olefins ranging from trimers to pentamers. The PAO structure is relatively regular with a uniform comb-shaped isoparaffin (IP) chain length of generally C_8_–C_10_. Based on the M_w_ of S_rt_ being 507 Da (Figure 2), the M_w_ of PAO could be predicted to be 520 Da, with a molecular formula of C_37_H_76_. Combined with the PAO comb-shaped structure, the preliminary estimated structural formula of PAO is shown in Figure 3.

As shown in Figure 2, M_n_ and M_w_ fluctuated as a function of temperature. M_n_ and M_w_ both decreased at 180 and 200 °C, with values of 503 and 494 Da, and 496 and 488 Da, respectively. The highest M_n_ and M_w_ values of 501 and 511 Da, respectively, were observed at 230 °C. Thereafter, M_n_ and M_w_ were again reduced and found to be stable at temperatures over 230 °C, with values of 495 and 487 Da, 494 and 486 Da, and 494 and 486 Da for S_250_, S_270_, and S_300_, respectively. There was insufficient thermal energy in the SHALOs to induce a larger degree of polymerization below 200 °C, at which the M_w_ was relatively low owing to the cracking degree being higher than the polymerization degree. In the range of 200–230 °C, increasing temperature caused larger-scale polymerization owing to TT, providing sufficient energy to significantly increase the M_w_. Therefore, M_n_ and M_w_ reached peak values in S_230_. The cracking degree was again higher than the polymerization degree when the temperature increased above 230 °C. In contrast, the SHALOs were less able to dissolve the polymers, resulting in coking and sludge formation at high temperatures. Under these experimental conditions, the M_w_ first decreased, then increased, and finally decreased again. The molecular weight distribution width index, D (D = M_w_/M_n_), varied from 1.018 to 1.020 depending on the TT temperature, showing that the oil samples were narrow heterogeneous dispersion systems at each temperature.

### 3.3. GC/MS Analysis

**Figure 4:**
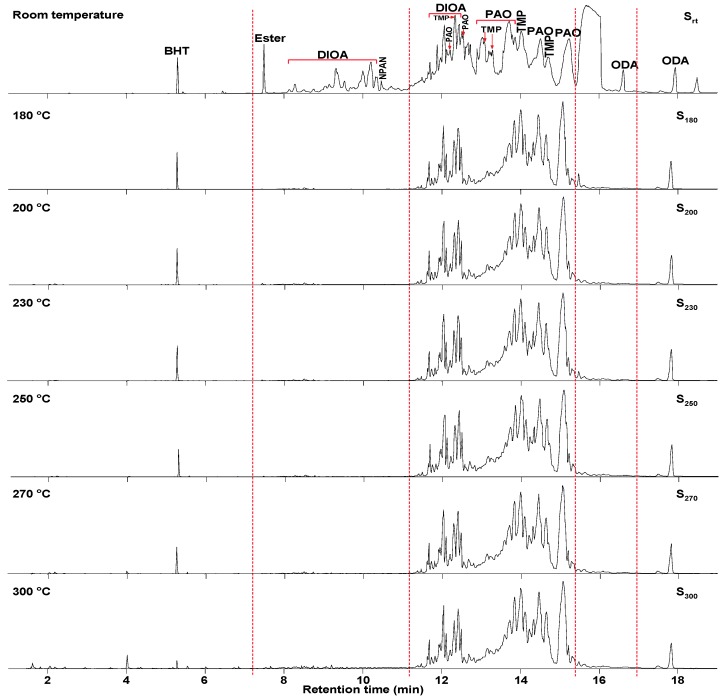
Total ion chromatograms (TIC) of the oil samples as a function of temperature.

As shown in Figure 4, S_rt_ was rich in two base oils (PAO and DIOA) and also contained four additives, three of which were antioxidants (butylated hydroxytoluene (BHT), N-phenyl-α-naphthylamine (NPAN), 4-octyl-N-(4-octylphenyl) aniline (ODA)), while the other was an antiwear additive (tris(3-methylphenyl) phosphate (TMP)).

Figure 4 also shows significant differences in oil composition as a function of TT temperature. In contrast to S_rt_, DIOA and NPAN were not detected in S_180_–S_300_ at retention times of 7–11 min and 15–17 min by GC/MS, respectively. Significant compositional differences existed between S_rt_ and S_180_, including a clear reduction in BHT, ODA, and TMP contents observed in S_180_. Furthermore, BHT and TMP were barely detected, together with an observed decrease in ODA in S_300_, implying that antioxidants were continuously removed from the system, preventing thermal cracking reactions. Furthermore, the compounds detected by GC/MS increased in intensity as a function of TT temperature over the first 11 min. The free radicals generated by thermal oxidation during the service life of lubricants are suggested to first be consumed by free-radical trapping antioxidant NPAN (medium-temperature antioxidant), which hinders more severe oxidation reactions [37] and, therefore, maintains the normal lubrication function of the oil.

**Figure 5:**
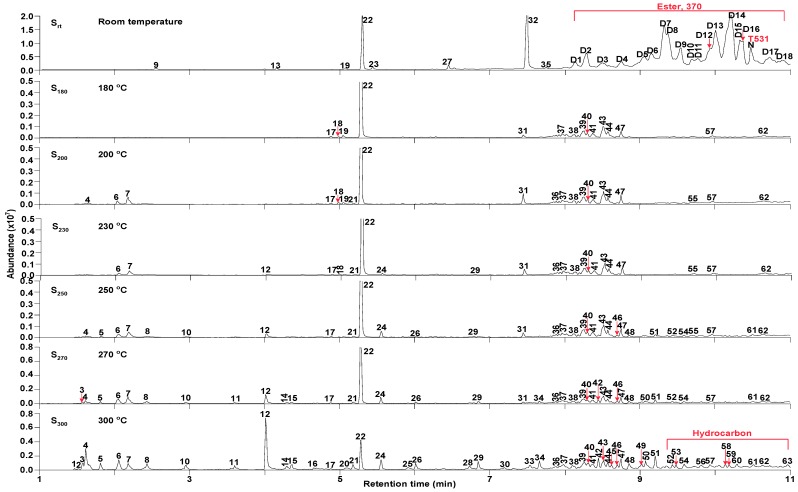
TICs of the oil samples as a function of temperature at retention times of 1–11 min.

Cracking reactions were performed with the oil samples according to the variation and relative contents (RCs) of small-molecule compounds. As shown in Figure 5 and Table 2, 82 organic compounds were detected by GC/MS, which were classified into n-alkanes (NAs), eight IPs, 21 OFs, 24 ETs, three alcohols (AHs), eight antioxygen and other compounds (AOs), and two unknowns (UKs).

Twenty-seven compounds were identified in S_rt_, including 23 ETs, three AOs, and one UK. Among them, ETs were the main components, accounting for the content of 10.90%. The three antioxidants were BHT (**22**), NPAN (N), and BHT derivatives (**19**). Fifteen compounds were detected in S_180_, including three NAs, two OFs, four IPs, one AH, and five AOs. The RCs of BHT (**22**), 2-, 6-, 10-, and 14-tetramethylhexadecane (**39**), and eicosane (**43**) accounted for a higher percentage. The distribution of compounds comprising S_200_ was similar to that of S_230_. Twenty-two compounds were detected in both samples, including seven NAs, two OFs, four IPs, two AHs, and six AOs. The RCs of 4-methylheptan-1-ol (**7**), BHT (**22**), 3,5-di-tert-butyl-4-hydroxybenzaldehyde, eicosane (**43**), and (E)-2-methylnonadec-7-ene (**47**) accounted for a higher percentage. Thirty-one compounds were detected in S_250_, including 12 NAs, eight OFs, four IPs, two AHs, and five AOs. The RCs of 4-methylheptan-1-ol (**7**), 2-(tert-butyl)-4-methylphenol (**12**), BHT (**22**), and the isomer of BHT (**24**) accounted for a higher percentage. Thirty-seven compounds were detected in S_270_, including 15 NAs, 11 OFs, four IPs, two AHs, and five AOs. The RCs of decane (**6**), 2-(tert-butyl)-4-methylphenol (**12**), BHT (**22**), and the isomer of **22** (**24**) accounted for a higher percentage. Fifty-two compounds were detected in S_300_, including 16 NAs, 21 OFs, and four AOs. The amounts of (Z)-oct-2-ene (4), 2-(tert-butyl)-4-methylphenol (**12**), the isomer of BHT (**24**), and NAs (C_9_–C_21_) had obviously increased. However, the BHT (**22**) content was drastically reduced (>50%) compared with that of S_rt_.

**Figure 6:**
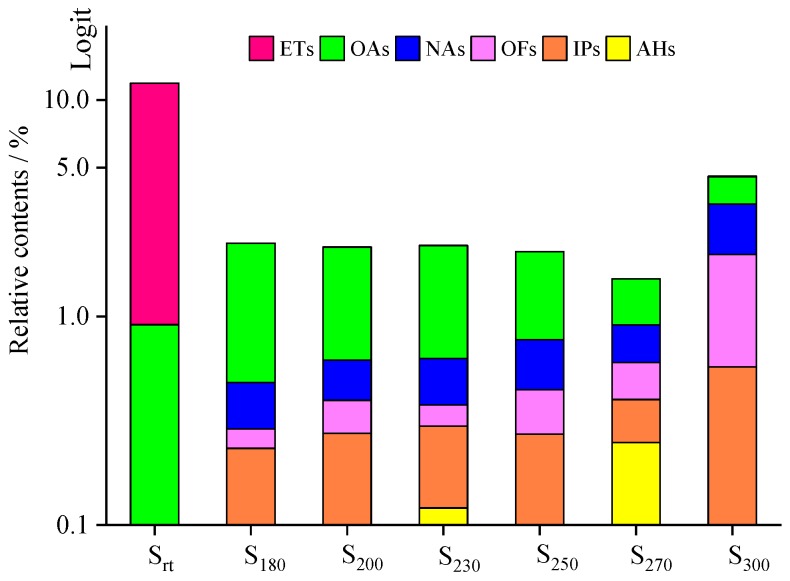
Distribution of group components in S_rt_ and S_180_–S_300_.

The above results showed that temperature played an important role in the compounds detected and influenced the compound type and RC. As shown in Figure 4 and Figure 5, at a retention time of 8–11 min, the RCs of DIOA and NPAN in S_rt_ were high, but the same compounds were not detected in S_180_. Instead, BHT underwent basic structure unit degradation to yield a small number of hydrocarbons. (Z)-Oct-2-ene (**4**, C_8_), NAs (**6**, C_10_; **21**, C_15_), and 4-methylheptan-1-ol (**7**, C_8_) were observed in S_200_, while the other two BHT derivatives, namely, 2-(tert-butyl)-4-methylphenol (**12**) and the isomer of BHT (**24**), were detected in S_230_. When the temperature reached 250 °C, small-molecule compounds showed increased contents, with the appearance of additional NAs (**5**, C_9_; **6**, C_10_; **8**, C_11_; **10**, C_12_; **21**, C_15_; **26**, C_16_). As shown in Figure 4 and Figure 6, when the temperature exceeded 270 °C, the amount and RC of small molecular compounds in S_300_ gradually increased compared with the other TT oil samples, reaching 52 compounds and 4.600%, respectively. This might be principally attributed to thermal cracking reactions of the base oils. Conversely, BHT, ODA, and TMP cracked only into relatively few small molecules through high-temperature oxidation. The results showed that the oil samples subjected to TT temperatures of >230 °C presented an increase in the number of small molecules, while the average molecular weight decreased, with concomitant deterioration in the properties. These observations were in agreement with the APC results.

## 4. Discussion

### 4.1. Physicochemical Properties: Changes with Temperature

**Figure 7:**
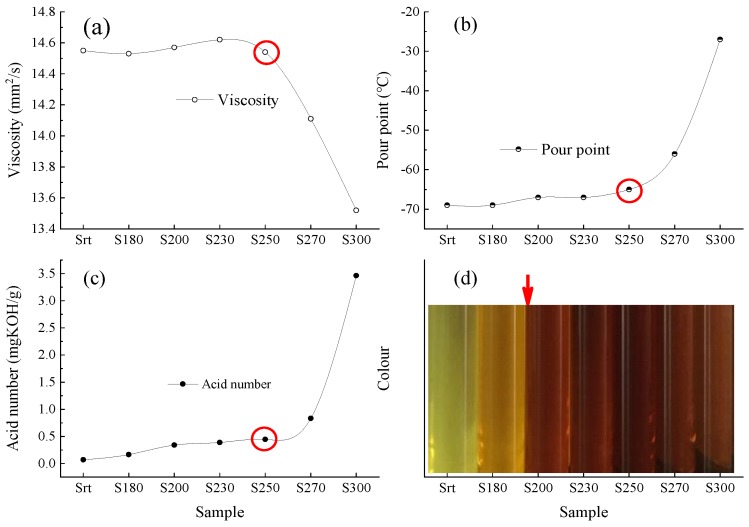
Kinematic viscosity at 40 °C, pour point, acid number, and color of the oil samples as a function of temperature. (**a**) Kinematic viscosity; (**b**) Pour point; (**c**) Acid number; (**d**) Colour.

As shown in Figure 7, the KV at 40 °C (ν_40_), PP, AN, and color showed different variational trends as a function of temperature. Changes in the trends of ν_40_, PP, and AN occurred at an inflection point of 250 °C. When the temperature was <250 °C, the ν_40_, PP, and AN values of the samples were relatively stable. The ranges of variation for ν_40_, PP, and AN were 14.6–14.4 mm^2^/s, −65 to −69 °C, and 0.07–0.45 mg KOH/g, respectively. When the temperature was >250 °C, the ν_40_, PP, and AN values of the samples changed dramatically. The ν_40_ values of S_270_ and S_300_ were reduced to 14.1 and 13.5 mm^2^/s, while the PP value was increased to −56 and −27 °C, and the AN value was increased to 0.8 and 3.5 mg KOH/g, respectively.

As shown in Figure 7, the inflection point for color was 200 °C. When subjected to TT temperatures of >200 °C, the oil samples were brown and black, while those at 180 °C were light brown. Compared with ν_40_, PP, and AN, the color was more sensitive to temperature, which induced rapid changes in the oil color. The color change preceded the degradation of the oil sample during the heating process and was relatively small during subsequent heating. Therefore, the color change should not be used as a basis for the degradation performance of the oil sample. Basing changes in the lubricating function of the oil solely on color changes is not ideal because the performance of the oil might not have actually deteriorated, resulting in waste. Studies have shown that changes in the oil color are related to the antioxidant effect of antioxidants [38]. The change in AN was derived from oxygen-containing compounds, such as alcohols, ketones, aldehydes, and acid esters, generated during high-temperature thermal oxidation [39]. PP is principally related to the α-methylene content in alkyl groups and aromatic or naphthenic rings. The NAs exhibit a higher PP, while the IPs exhibit a lower PP [40]. During the low- and medium-temperature stages (<250 °C), the antioxidant effect, while changing the color of the oil samples, also prevented the cracking of the PAO and DIOA molecular chains in the oil samples and, to a certain extent, the formation of other substances [41]. Therefore, the degradation rate of ν_40_, PP, and AN at this stage was reduced. At the high-temperature stage (>250 °C), the molecular structure of PAO was destroyed, and the type and content of n-alkanes exceeded that of o-alkanes, leading to an increase in PP [42]. Simultaneously, with the decrease in antioxidant content, the antioxidant effect was obviously weakened. The oil samples were typically subjected to high-temperature thermal oxidation and cracking, and the contents of small molecular oxygen-containing compounds, such as alcohols, ketones, aldehydes, and acid esters, significantly increased, leading to an increase in AN.

The KV of the lubricating oil is mainly derived from the interaction force between molecules. The distance between the molecules increases with rising temperature owing to volume expansion, which results in the decrease in force between molecules and, therefore, a decrease in viscosity. The small molecular compounds produced by cracking at high temperatures have made a greater contribution to the reduction in KV [43]. Based on the APC results, at >230 °C, the temperature was known to rapidly decrease both M_n_ and M_w_. Therefore, the relative number of small-molecule compounds increased, which led to a decrease in ν_40_ at high temperatures.

### 4.2. Molecular Structure: Changes with Temperature

**Figure 8:**
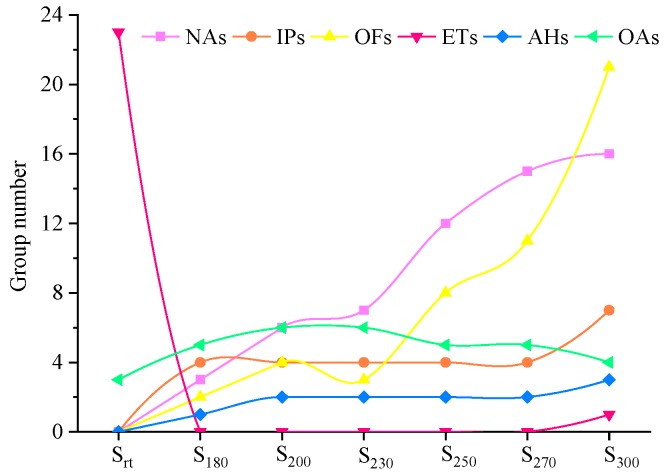
Group component numbers in S_rt_ and S_180_–S_300_.

The molecular structure of SLOs determines the thermal oxidation stability when oils are exposed to harsh operating conditions [2,44]. As shown in Figure 6 and Figure 8, there were significant differences in the species and RCs of compounds detected by GC/MS between the fresh oil and aged oils. The fresh oil (S_rt_) comprised 23 ETs (10.90%) and three AOs (0.913%), while hydrocarbons, alcohols, and AO compounds were the main compounds observed in the aged oils (S_180_–S_300_). ETs were not detected in S_180_–S_270_, but pentylpentanoate was observed in S_300_. Degradation products were produced during the test, implying that the SHALO thermal oxidation process was highly complex. Meanwhile, any small changes in the experimental conditions, such as the experimental reactor, heating and cooling conditions, sampling approaches, and production batches of the same lubricating oils, could affect the results and the changing trend under high temperatures.

**Figure 9:**
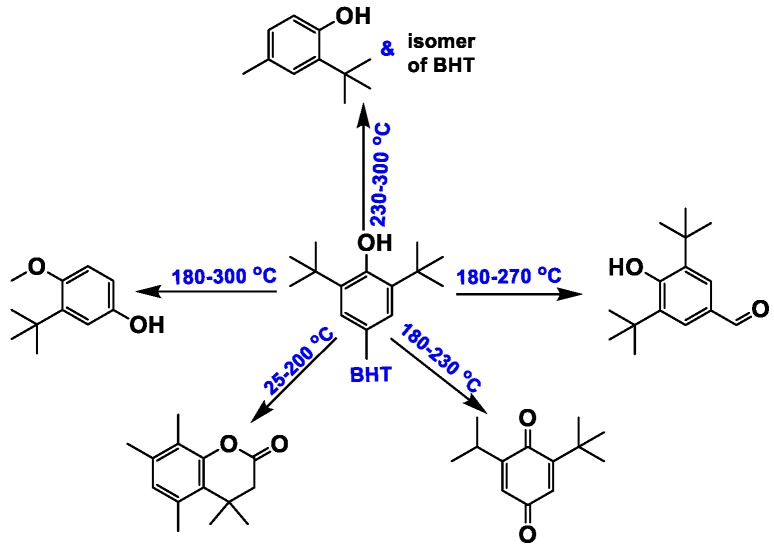
Butylated hydroxytoluene (BHT) derivative distributions.

**Figure 10:**
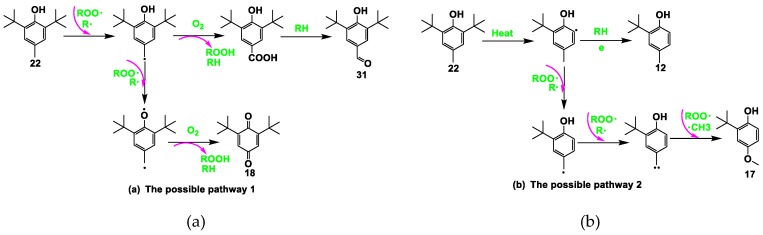
Possible reaction pathways of BHT. (**a**)The possible pathway 1; (**b**) The possible pathway 2.

The oxidation process of lubricating oils has three stages [26]. First, the antioxidant and antiwear additives are consumed, followed by oxidative degradation of the base oil, and, finally, polymerization, which leads to an increase in the oil viscosity in the final stage. The APC and GC/MS data were in agreement with the three stages of oxidation. The additives were depleted at 180 °C in the first stage. The RCs of the three antioxidants (BHT, NPAN, and ODA) and one antiwear additive (TMP) were reduced, but the amount of these compounds increased. Four derivatives of BHT are shown in Figure 9, and the number of carbon atoms associated with the NAs in S_180_ was not less than 20. Generally, small-molecule compounds, which increased in content during degradation of the lubricating oil, resulted in decreased M_n_ and M_w_ values, while cross-linking and polycondensation of the compounds resulted in increased M_n_ and M_w_ values. Furthermore, decane was detected in S_180_. The oil samples at <200 °C were concluded to be partially pyrolyzed, as characterized by a growing amount of small-molecule compounds compared with that of macromolecules. The KV values of S_180_ and S_200_ were relatively low, suggesting that the formation of hydroperoxides in the initial oxidation period seriously deteriorated the properties of the aging oils. In the temperature range of 200–230 °C, the degree of polycondensation was greater than that of cracking in the aged oils owing to a growing volume of macromolecular compounds, while the KV of S_230_ was observed to increase [45,46,47]. During this stage, the formation of polar organic compounds in the oil (such as ketones, alcohols, carboxylic acids, and esters) was more pronounced, with 4-methylheptan-1-ol and 2-(hexadecyloxy)ethan-1-ol detected. Furthermore, two new derivatives of BHT—2-(tert-butyl)-4-methylphenol and the isomer of BHT—were present in S_230_. The possible reaction phenolic hydroxyl oxygen pathways of BHT are shown in Figure 10. When heated, BHT acted as a radical terminator. The phenolic hydroxyl oxygen could participate in the benzene conjugated system to induce electronegativity, with the pushing of electron density from the o-, m-, and p-substituents resulting in a reduction of O–H polarity. Hydrogen atoms were quickly abstracted by the free radicals of the lubricating oils to improve the thermal oxidation stability. The change in the composition of the SHALO was similar to the three-stage oxidation process (depletion of additives, degradation of the base oil, and polymerization) when the TT temperature was <230 °C. In the temperature range of 250–300 °C, the degree of thermal cracking in the oil sample was greater than that of thermal polycondensation, characterized by a growing amount of small-molecule compounds. C_10_–C_18_ compounds were detected in S_250_–S_300_, and the RC of BHT derivative 2-(tert-butyl)-4-methylphenol increased from 0.009% in S_230_ to 0.646% in S_300_. Therefore, the KV of the aged oils was also significantly reduced [2,13].

**Figure 11:**
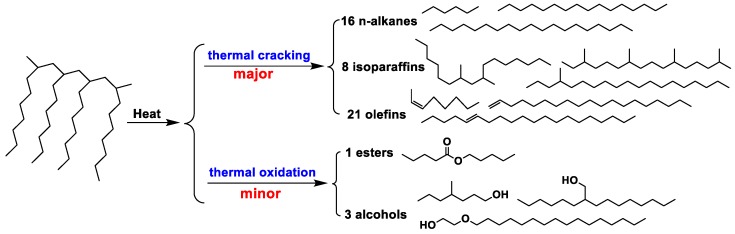
Possible reaction types of PAO.

Overall, the polycondensation and cracking reactions of the oil samples were shown to occur simultaneously at high temperatures, with thermal cracking as the predominant mechanism of these reactions (Figure 11). There was a significant difference between the degree of polycondensation and cracking reactions as a function of temperature. After polycondensation and pyrolysis reactions, the physicochemical properties of the oil samples changed, with the thermal stability decreasing to the degree that resulted in the deterioration of these properties. The results showed that at temperatures of <250 °C, the compounds in the base oil were slightly cracked, and the antioxidants significantly changed. Polycondensation is the predominant reaction below 250 °C, which might explain why lower wear was observed for the aged oils compared with fresh oil, and why the films formed from the aged oils provided superior wear performance compared with that of the film formed from fresh oil [24]. When the temperature exceeded 250 °C, the compounds present in the oil samples were mainly pyrolyzed, but the antioxidant effect was less obvious. Furthermore, the types of small-molecule compounds increased, and a large number of small-molecule compounds, such as OFs and NAs, appeared. There were a large number of small molecular compounds, which indicated that a significant degree of thermal oxidation cracking occurred in the lubricating oil. Therefore, the molecular weight exhibited a downward trend, the viscosity decreased, and the oil film was not able to reach a certain thickness (≤0.01 μm) [16]. The lubricating function of the lubricating oil was significantly impacted, with friction and wear occurring, leading to the deterioration of the oil properties.

### 4.3. CA of the Oil Samples

Clustering aims to seek natural groupings in the data based on the similarity of individual components or objects, such that the objects of the same group, or cluster, are more similar than different groups [48,49,50]. CA is considered a principal task of data exploration, is a common technique for statistical analysis and chemometric approaches, and is widely used in molecular spectroscopy [51,52,53]. The results of CA were intended to elucidate the compound structure and compositional changes of SHALOs resulting from oxidative high-temperature degradation, allowing detection of the degradation stages of SHALOs and signaling of the need for an oil change when warranted by the oil conditions [54]. Furthermore, the SHALO clusters could be used to detect possible relationships between compound structure and compositional changes at the molecular level and, therefore, facilitate the discovery of physicochemical properties at the macro level.

**Figure 12:**
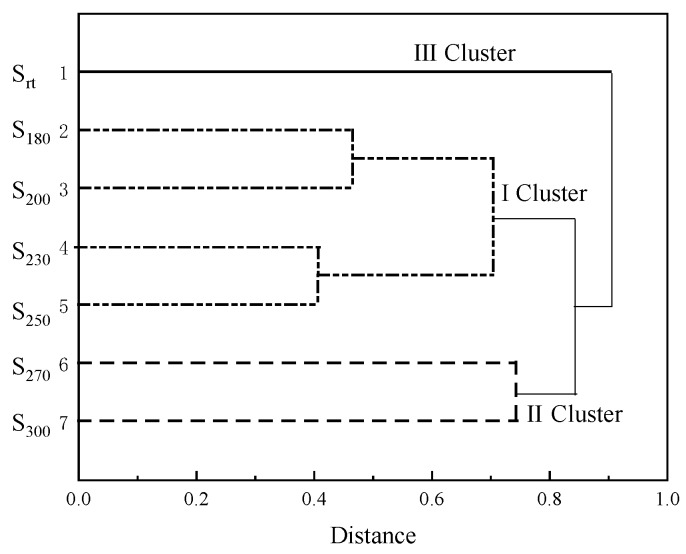
Dendrogram of the oil samples by cluster analysis based on the average linkage and Pearson correlation coefficients.

Based on 82 compounds detected from seven samples by GC/MS at retention times of 1–11 min, the SHALO clusters were classified by CA using Origin 9.0 software. The average distance between groups, data standardization, and the Pearson correlation coefficient was chosen for hierarchical CA. As shown in Figure 12, the system CA tree diagram showed that seven types of oils could be classified, as follows: S_180_, S_200_, S_230_, and S_250_ belonging to cluster I, S_270_ and S_300_ belonging to cluster II, and S_rt_ belonging to cluster III. The classifications of clusters I and II were consistent with the inflection points of the M_n_, M_w_, ν_40_, PP, and AN values of the oil samples at 250 °C. The classification of S_rt_ into cluster III was also consistent with the physical system studied. CA results at the molecular level were observed to be in good agreement with the changes observed to the physicochemical properties of the samples on the macro level. Gong et al. observed that hierarchical CA results based on two-channel and differential dielectric spectroscopy (TD-DES) data were also in good agreement with those based on FT-IR data and proposed using the TD-DES technique to monitor the oil and conduct hierarchical CA [54]. Furthermore, the present study offered a platform for further researching the molecular mechanism of oil sample degradation performance.

## 5. Conclusions

In this study, the compositional and structural characteristics of a SHALO were clarified under treatment at different temperatures through compound identification and analysis. The data provided scientific validation and technical support for molecular mechanisms related to the physicochemical properties and degree of SHALO deterioration as a function of temperature. Twenty functional groups, including comb-like alkanes, long-chain diesters, amines, and phenols, were identified in the SHALO. The antioxidants induced obvious effects that hindered the formation of free radicals, and NPAN was exhausted when the temperature was higher than 180 °C. Therefore, the cracking reactions were slower compared with polycondensation at low and medium temperatures (<250 °C). However, at high temperatures (>250 °C), the role of the antioxidants gradually weakened, which significantly promoted the cracking reactions. The number and relative contents of small-molecule compounds in S_300_, mainly composed of NAs, IPs, OFs, and AHs, were 52 and 4.600%, which were significantly higher compared with those in S_180_ (15 and 2.228%). A correlation existed between the rapid degradation of physicochemical properties (such as color, KV, PP, and AN) and the rapid increase in small-molecule compounds under high-temperature treatment. CA, based on the RCs of compounds, was in good agreement with the classification based on macroscopic physicochemical properties, which revealed the molecular mechanism of the degradation in properties at high TT temperatures. Furthermore, on this basis, the high-temperature SHALO performance would be improved, and the service life prolonged by developing chemical analysis.

## Figures and Tables

**Table 1:** FT-IR spectra assignments for S_rt_, S_180_, S_200_, S_230_, S_250_, S_270_, and S_300_.

Type	Group	Characteristic Frequency/cm^−1^	Intensity∗	Belonging
alkyl group	–CH_3_	2957	vs	ν^as^
	–CH_2_–	2925	vs	ν^as^
	–CH_2_–	2854	vs	ν^s^
	–CH_2_–	1465	vs	δ
	–CH(CH_3_)_2_	1738	s	δ^as^
	–(CH_2_)_n_–	721	m	δ
aldehyde group	C–H	2730	w	ν_CH_
	C–H	2672	w	γ
ester	C=O	1741	vs	ν_C=O_
	C–O–C in long-chain saturated fatty acids	1169 and 1142	m	ν^as^_(C_–_O_–_C)_
benzene	framework vibrations	1611 and 1588	w	ν
	o-	879	m	γ_=C–H_
	m-	821	m	
	p-	779	m	
olefin	C=C	1640	vw	ν_C=C_
	=CH–H	970	s	ω_C=C_
secondary amine	–NH	1506	w	ν_NH_
	C-N in aromatic compounds	1241 and 1314	w	ν_C–N_
phenol	–OH	3645	w	ν_OH_
	O–H	686	w	τ

∗ vs—very strong; s—strong; m—medium; w—weak; vw—very weak.

**Table 2:** Compounds detected in the total ion chromatograms of the oil samples as a function of temperature at retention times of 1–11 min.

Peak	Retention Time (min)	Compounds	Chemical Formula	Molecular Weight	Structural Formula	Relative Content (%)						
						S_rt_	S_180_	S_200_	S_230_	S_250_	S_270_	S_300_
**1**	1.468	1-Butene	C_4_H_8_	56	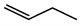	-	-	-	-	-	-	0.015
**2**	1.518	Hexane	C_6_H_14_	86	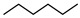	-	-	-	-	-	-	0.012
**3**	1.579	3-methylhept-1-ene	C_8_H_16_	112	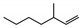	-	-	-	-	-	0.006	0.094
**4**	1.615	(Z)-oct-2-ene	C_8_H_16_	112	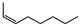	-	-	0.018	-	0.011	0.016	0.163
**5**	1.814	Nonane	C_9_H_20_	128	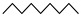	-	-	-	-	0.011	0.013	0.084
**6**	2.057	Decane	C_10_H_22_	142	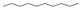	-	-	0.028	0.024	0.044	0.042	0.168
**7**	2.182	4-methylheptan-1-ol	C_8_H_18_O	130	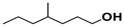	-	-	0.086	0.066	0.087	0.058	0.122
**8**	2.432	Undecane	C_11_H_24_	156	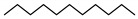	-	-	-	-	0.024	0.021	0.12
**9**	2.547	2-ethylhexyl pentanoate	C_13_H_26_O_2_	214	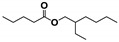	0.024	-	-	-	-	-	-
**10**	2.948	Dodecane	C_12_H_26_	170	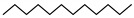	-	-	-	-	0.009	0.01	0.083
**11**	3.601	Tridecane	C_13_H_28_	184	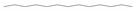	-	-	-	-	-	0.007	0.027
**12**	4.015	2-(tert-butyl)-4-methylphenol	C_11_H_16_O	164	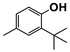	-	-	-	0.009	0.038	0.059	0.646
**13**	4.157	2-ethylhexyl hexanoate	C_14_H_28_O_2_	228	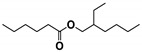	0.013	-	-	-	-	-	-
**14**	4.295	(E)-tetradec-5-ene	C_14_H_28_	196	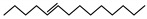	-	-	-	-	-	0.008	0.063
**15**	4.358	Tetradecane	C_14_H_30_	198	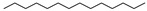	-	-	-	-	-	0.009	0.086
**16**	4.656	pentylpentanoate	C_10_H_20_O_2_	172	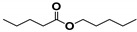	-	-	-	-	-	-	0.018
**17**	4.883	3-(tert-butyl)-4-methoxyphenol	C_11_H_16_O_2_	180	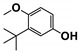	-	0.009	0.008	0.005	0.008	0.005	0.013
**18**	4.981	2,6-di-tert-butylcyclohexa-2,5-diene-1,4-dione	C_14_H_20_O_2_	220	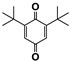	-	0.005	0.012	0.005	-	-	-
**19**	5.064	4,4,5,7,8-pentamethylchroman-2-one	C_14_H_18_O_2_	218	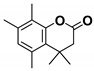	0.014	0.019	0.016	-	-	-	-
**20**	5.096	(E)-tetradec-5-ene	C_14_H_28_	196	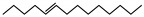	-	-	-	-	-	-	0.055
**21**	5.175	Pentadecane	C_15_H_32_	212	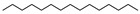	-	-	0.007	0.005	0.01	0.007	0.083
**22**	5.281	2,6-di-tert-butyl-4-methylphenol	C_15_H_24_O	220	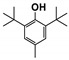	0.833	1.692	1.402	1.472	1.13	0.492	0.363
**23**	5.434	2-methylbutyl heptanoate	C_12_H_24_O_2_	200	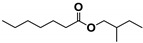	0.049	-	-	-	-	-	-
**24**	5.550	isomer of 22	C_15_H_24_O	220	-	-	-	-	0.002	0.043	0.03	0.123
**25**	5.909	(Z)-hexadec-7-ene	C_16_H_32_	224	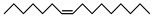	-	-	-	-	-	-	0.042
**26**	6.013	Hexadecane	C_16_H_34_	226	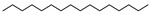	-	-	-	-	0.004	0.011	0.085
**27**	6.446	2-ethylhexyl 2-methoxyacetate	C_11_H_22_O_3_	202	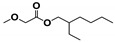	0.064	-	-	-	-	-	-
**28**	6.729	(E)-heptadec-8-ene	C_17_H_34_	238	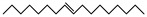	-	-	-	-	-	-	0.062
**29**	6.846	Heptadecane	C_17_H_36_	240	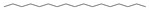	-	-	-	0.006	0.009	0.017	0.112
**30**	7.192	7,9-dimethylhexadecane	C_18_H_38_	254	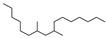	-	-	-	-	-	-	0.017
**31**	7.443	3,5-di-tert-butyl-4-hydroxybenzaldehyde	C_15_H_22_O_2_	234	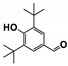	-	0.019	0.075	0.048	0.035	0.013	-
**32**	7.493	2-ethylhexyl isobutyl carbonate	C_13_H_26_O_3_	230	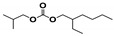	1.427	-	-	-	-	-	-
**33**	7.534	(E)-octadec-9-ene	C_18_H_36_	252	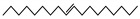	-	-	-	-	-	-	0.053
**34**	7.661	Octadecane	C_18_H_38_	254	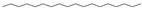	-	-	-	-	-	0.016	0.096
**35**	7.747	unknown	-	-	-	0.021	-	-	-	-	-	-
**36**	7.880	(Z)-nonadec-5-ene	C_19_H_38_	266	-	-	-	0.011	0.007	0.009	0.017	-
**37**	7.979	10-methylnonadecane	C_20_H_42_	282	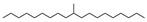	-	0.007	0.02	0.022	0.022	0.025	0.128
**38**	8.099	nonadec-1-ene	C_19_H_38_	266	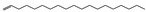	-	0.022	0.032	0.016	0.03	0.023	0.067
**39**	8.259	2,6,10,14-tetramethylhexadecane	C_20_H_42_	282	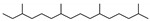	-	0.007	0.083	0.069	0.081	0.063	0.113
**40**	8.310	2-(hexadecyloxy)ethan-1-ol	C_18_H_38_O_2_	286	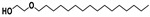	-	0.022	0.028	0.027	0.034	0.021	0.072
**41**	8.367	4-methylnonadecane	C_20_H_42_	282	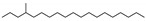	-	0.05	0.05	0.051	0.048	0.03	0.087
**42**	8.445	Nonadecane	C_19_H_40_	268	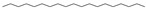	-	-	-	-	-	0.02	0.103
**43**	8.514	Eicosane	C_20_H_42_	282	-	-	0.155	0.15	0.15	0.128	0.075	0.116
**44**	8.571	2,3-dimethylnonadecane	C_21_H_44_	296	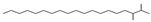	-	0.065	0.067	0.036	0.043	0.034	0.057
**45**	8.620	2-hexyldecan-1-ol	C_16_H_34_O	242	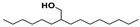	-	-	-	-	-	-	0.055
**46**	8.697	unknown	-	-	-	-	-	-	-	0.01	0.019	0.042
**47**	8.745	(E)-2-methylnonadec-7-ene	C_20_H_40_	280	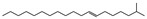	-	0.048	0.058	0.055	0.06	0.039	0.116
**48**	8.854	icos-1-ene	C_20_H_40_	280	-	-	-	-	-	0.017	0.018	0.085
**49**	9.022	(E)-icos-9-ene	C_20_H_40_	280	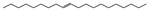	-	-	-	-	-	-	0.076
**50**	9.073	(E)-icos-5-ene	C_20_H_40_	280	-	-	-	-	-	-	0.03	0.123
**51**	9.203	Heneicosane	C_21_H_44_	296	-	-	-	-	-	0.014	0.032	0.152
**52**	9.431	olefin (C ≥ 21)	-	-	-	-	-	-	-	0.016	0.023	0.04
**53**	9.481	isoparaffin (C ≥ 21)				-	-	-	-	-	-	0.047
**54**	9.589	olefin (C ≥ 21)				-	-	-	-	0.015	0.022	0.072
**55**	9.690	isoparaffin (C ≥ 21)				-	-	0.017	0.019	0.024	-	-
**56**	9.821	olefin (C ≥ 21)				-	-	-	-	-	-	0.04
**57**	9.936	normal alkane (C ≥ 21)				-	0.024	0.02	0.023	0.031	0.026	0.064
**58**	10.137	olefin (C ≥ 21)				-	-	-	-	-	-	0.037
**59**	10.188	isoparaffin (C ≥ 21)				-	-	-	-	-	-	0.032
**60**	10.291	olefin (C ≥ 21)				-	-	-	-	-	-	0.053
**61**	10.511	olefin (C ≥ 21)				-	-	-	-	0.015	-	0.062
**62**	10.631	normal alkane (C ≥ 21)				-	0.015	-	0.027	0.021	-	0.033
**63**	10.967	olefin (C ≥ 21)				-	-	-	-	-	-	0.077
**D1–D18**	8.135–10.893	dioctylhexanedioate	C_22_H_42_O_4_	370	-	10.155	-	-	-	-	-	-
**N**	10.549	N-phenylnaphthalen-1-amine	C_16_H_13_N	219	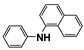	0.450	-	-	-	-	-	-
